# An urgent need for COP27: confronting converging crises

**DOI:** 10.1007/s11625-022-01253-5

**Published:** 2022-11-10

**Authors:** Jim Falk, Rita R. Colwell, Swadhin K. Behera, Adel S. El-Beltagy, Peter H. Gleick, Charles F. Kennel, Yuan Tseh Lee, Cherry A. Murray, Ismail Serageldin, Kazuhiko Takeuchi, Tetsuzo Yasunari, Chiho Watanabe, Joanne Kauffman, Kurt Soderland, Ismahane Elouafi, Raj Paroda, Ashok K. Chapagain, John Rundle, Naota Hanasaki, Haruo Hayashi, Ebun Akinsete, Sachiko Hayashida

**Affiliations:** 1 School of Geography, Earth and Atmospheric Sciences, University of Melbourne, Melbourne, Australia; 2 University of Wollongong, Wollongong, Australia; 3 Center for Bioinformatics and Computational Biology, University of Maryland, College Park, USA; 4 Johns Hopkins Bloomberg School of Public Health, Baltimore, USA; 5 Application Laboratory, Japan Agency for Marine-Earth Science and Technology (JAMSTEC), Yokosuka, Japan; 6 Department of Ocean Technology, Policy and Environment, The University of Tokyo, Tokyo, Japan; 7 International Dryland Development Commission, Arid Land Agricultural Graduate Studies and Research Institute, Ain Shams University, Cairo, Egypt; 8 Pacific Institute for Studies in Development, Environment and Security, Oakland, USA; 9 Scripps Institution of Oceanography, University of California, San Diego (UCSD), San Diego, USA; 10 Centre for Science and Policy, University of Cambridge, Cambridge, UK; 11 Academia Sinica, New Taipei, Taiwan; 12 Harvard University, Cambridge, USA; 13 University of Arizona, Tucson, USA; 14 The Library of Alexandria, Alexandria, Egypt; 15 Institute for Global Environmental Strategies (IGES), Kanagawa, Japan; 16 Institute for Future Initiatives (IFI), The University of Tokyo, Tokyo, Japan; 17 RIHN Center, Research Institute for Humanity and Nature (RIHN), Kyoto, Japan; 18 Kyoto Climate Change Adaptation Center (KCCAC), Kyoto, Japan; 19 School of Tropical Medicine and Global Health, Nagasaki University, Nagasaki, Japan; 20 The University of Tokyo, Tokyo, Japan; 21 Science for Sustainable Societies, Springer-Verlag, Paris, France; 22 Safe Water Network, New York, USA; 23 Food and Agriculture Organisation of the United Nations (FAO), Rome, Italy; 24 Trust for Advancement of Agricultural Sciences (TAAS), New Delhi, India; 25 Pacific Institute, London, UK; 26 Department of Physics and Astronomy, University of California, Davis, Davis, USA; 27 Center for Climate Change Adaptation (Climate Change Impacts Assessment Research Section), National Institute of Environmental Studies, Tsukuba, Japan; 28 National Research Institute for Earth Science and Disaster Resilience, Ibaraki, Japan; 29 International Centre for Research on the Environment and the Economy/UN Sustainable Development Solutions Network Greece, Athens, Greece; 30 Research Institute for Humanity and Nature Faculty of Science (RIHN), Kyoto, Japan

**Keywords:** Climate Change, Biodiversity, Food and Water crisis, Risk interaction

## Abstract

The last 12 months have provided further evidence of the potential for cascading ecological and socio-political crises that were warned of 12 months ago. Then a consensus statement from the Regional Action on Climate Change Symposium warned: “the Earth’s climatic, ecological, and human systems are converging towards a crisis that threatens to engulf global civilization within the lifetimes of children now living.” Since then, the consequences of a broad set of extreme climate events (notably droughts, floods, and fires) have been compounded by interaction with impacts from multiple pandemics (including COVID-19 and cholera) and the Russia–Ukraine war. As a result, new connections are becoming visible between climate change and human health, large vulnerable populations are experiencing food crises, climate refugees are on the move, and the risks of water, food, and climate disruption have been visibly converging and compounding. Many vulnerable populations now face serious challenges to adapt. In light of these trends, this year, RACC identifies a range of measures to be taken at global and regional levels to bolster the resilience of these populations in the face of such emerging crises. In particular, at all scales, there is a need for globally available local data, reliable analytic techniques, community capacity to plan adaptation strategies, and the resources (scientific, technical, cultural, and economic) to implement them. To date, the rate of growth of the support for climate change resilience lags behind the rapid growth of cascading and converging risks. As an urgent message to COP27, it is proposed that the time is now right to devote much greater emphasis, global funding, and support to the increasing adaptation needs of vulnerable populations.

The last 12 months have provided further evidence of the potential for cascading ecological and socio-political crises that some of us warned of 12 months ago (Falk et al. 2021). At that time, we said (Falk et al. 2022): “the Earth’s climatic, ecological, and human systems are converging towards a crisis that threatens to engulf global civilisation within the lifetimes of children now living.”

Drivers of this developing crisis include climate change and degradation of biodiversity in the context of globally inadequate health infrastructures, growing conflicts and stark inequalities. Climate change will impact agriculture and ecosystems when at their most vulnerable during what is predicted to be peak human population (around 2090). Just when the world is most crowded is when these combined risks may well be largest and their consequences most likely to interact.

There has been no slowdown in global warming. Reductions in greenhouse gas emissions lag further behind the commitments made in the 2015 Paris Agreement. The effects are already visible (Pörtner et al. 2022). This year additional evidence identifies converging risk mechanisms leading to compounding and cascading impacts. Consequently, security and sustainability risks are growing.

For example:

- *New connections are emerging between climate change impacts and human health*: The world is currently experiencing three pandemics—two ongoing: COVID-19 and cholera (of which there are millions of cases each year), and monkeypox. New more highly infective variants of COVID-19 have evolved, creating major health challenges especially in economically vulnerable countries. Satellite data are now showing a clear relationship between warming climate and the water-borne diseases carried by zooplankton (Brumfield et al. 2022), exacerbating humanity’s pandemic stress.
- *Large vulnerable populations are already experiencing food crises*: Despite the world’s riches, millions of people still suffer from inadequate nutrition. Some 3.2 billion people were food insecure last year with up to 828 million facing hunger (FAO et al. 2022). Those numbers are increasing, especially in Africa. Food systems are highly climate dependent and are being hit hard by climate extremes, geopolitical conflicts, and COVID-19-related economic disruptions, all of which contribute to worsening in food insecurity.
- *The risks of water, food, and climate disruption are converging*: As the planet warms, dryer areas are becoming dryer. Wetter areas are becoming wetter. This will impact crop production, especially in some of the most populated and poorest regions. Water connects everything from energy generation to forestry and food to ecological and human health. But there is growing evidence that the planet’s hydrological systems are already shifting in response to climate change, worsening the mismatch between water supply and demand for food. The “green revolution”—the growth in food productivity to feed an expanding population—has been founded on massive use of irrigation using groundwater, of which some 30–40% is non-renewable (Bierkens et al. 2019), and is being extracted at increasingly unsustainable rates. As water levels drop, pumping costs and energy demands rise.
  In addition, countries in the dry area (such as Egypt, Jordan, and Morocco) will suffer from acute water poverty. The green revolution may in the long term seem not so much to have solved the potential world food crisis as to have delayed it. In the highly populated and vulnerable populations of the planet, food is running short not because the world does not grow enough, but because of poverty, conflict and inequitable food distribution. Whilst in the past such risks have been dealt with separately, they are inter-related and must be dealt with jointly.
- *Flows of environmental refugees are already evident and likely to increase as pressures on vulnerable regions grow*: It is estimated that between 200 million and 1.2 billion environmental refugees may be on the move by 2050 (Ida 2021), creating significant social, economic and political pressures across different regions. Such stresses increase the vulnerability of populations to other stresses. With greater vulnerability comes a reduced resilience to new destabilisations (such as those created as an outcome of the Russia–Ukraine war (National Academies of Sciences, Engineering, and Medicine 2022).

## Moving forward

Previously, international attention and investment have focussed primarily on the challenges of mitigation of climate change. The compounding interactions of converging risks heighten the possibilities of “tipping points”. This adds greater weight to the need to build resilience, especially for the densely populated and economically disadvantaged populations who do not have the resources to cope. It is vital to provide the capacity to assess and monitor these risks, anticipate impacts, and build resilience to them.

In short, there are increasingly serious impacts that mitigation cannot avoid. It is time to recognise that greater support should be given to adaptation. Neither the ability nor resources to adapt are evenly distributed around the world. Often those who have contributed least to the developing crisis suffer most from its effects. Increased international financial, institutional and investment support are needed to address such inequalities.

Effective climate change mitigation strategies depend on vulnerable populations being sufficiently resilient to climate impacts to be able to invest in their transition to a net zero emissions economy. The global community has failed to deliver on undertakings made over the last decade to bring significant resources to developing regions for the task of adaptation (including the promised $100 billion). This failure should be rectified, and the size of commitments greatly increased.

The UN’s adoption of the Sendai Framework for Disaster Risk Reduction (UNDRR 2015), and the Sustainable Development Goals (UN General Assembly 2015), provide a useful global framework and set of targets on how to build resilience. However, to be effective, implementation efforts require strong collaboration and full implementation by all nation states in a coherent integrated way with comparable levels of investment.

Urgency repeatedly expressed by scientists, and the international mandates promised at meeting after meeting, have proven insufficient to inspire sufficient actions at local levels. Successfully building resilience requires people to recognise their interconnectedness with nature and each other. It also requires consistent input from the most vulnerable (notably women speaking also for their children). As the Jenna Declaration (UNESCO 2021) spells out, sensitivity to these cultural dimensions need to be embedded in strategy, policy making, research and education (Werlen et al. 2019).

Over its 14 years, RACC has advocated the need to build “knowledge-action networks” that can tailor knowledge from the natural and social sciences to specific adaptation needs as identified by local communities. The UN Sustainable Development Solutions Network (https://www.unsdsn.org), which focuses on the implementation of the SDGs, can play a constructive role in this area.

Water systems are vulnerable to climate and other shocks. The resilience of these systems needs to be expanded in the face of uncertainty. Water systems can be improved and water use can become more equitable and efficient. In 2020, the United Nations estimated some 2 billion people do not have access to safely managed water free from contamination (UNICEF and WHO 2021). Work by the Safe Water Network (https://safewaternetwork.org) shows how an approach linking leading technical knowledge with local engagement can create access to reliable, equitable, and sustainable water supply.

A wide range of technical assistance is needed, from biotechnology (for example, development of crop varieties that are resilient to regional climate changes) to applications of IT, AI, and renewable energy and storage (El-Beltagy 2019). Africa produces only a fraction of the food output it could with appropriate assistance (Goedde et al. 2019). At the same time, local communities need to participate in assessing risks in the context of local vulnerabilities and in selecting which tools may be most helpful to reduce those vulnerabilities.

At the regional and national levels, agriculture—which with forestry, land use and food processing represents some 30% of carbon emissions (Shukla et al. 2019)—can play a key role not only as a source but also a key part of the solution to climate change (notably through soil and water management and carbon sequestration).

Adaptation requires change to agricultural and food systems. As proposed at the RACC, co-sponsored Egypt-based Drylands Webinar (IDDC et al. 2022) economic and other measures to assist the most vulnerable are required. Food needs to be grown with greater water efficiency. Food waste—some 14% before retail (FAO 2019), and 17% of what is then sold (UNEP 2021)—needs to be significantly reduced. Climate Smart Agriculture should be a national priority with increased R&D funding for scaling innovations around adaptation and mitigation. This requires integrated government action as well as strong engagement and support at the community level. It should be combined with educational efforts to enrich understanding of the human–nature relationship, and blend contemporary scientific approaches with traditional knowledge of the environment (Rafea 2011, 2019).

Integrated thinking also means that comprehensive assessment methodologies need to be developed to prevent responses to one set of risks worsening others (Fujimori 2020). Examples of this danger include: increased energy demand from desalination plants in arid regions; more widespread fossil fuel-powered air-conditioning in the face of worsening heat waves; and pressure on agricultural land and increased degradation of biodiversity from production of biofuels, notably in the context of the BECCS (Bio-energy with Carbon Capture and Storage).

State of the art early warning systems should be developed, tailored to the needs of vulnerable regions and able to predict threats to crop production several seasons ahead, with support for small-scale and subsistence farmers who are most vulnerable to these impacts to utilise such predictions. We stress that at the local level, many initiatives by communities have been extremely innovative and effective in building resilience. These community efforts need to be supported and encouraged.

In summary, at all scales, adaptation requires globally available local data, reliable analytic techniques, community capacity to plan adaptation strategies, and the resources (scientific, technical, cultural, and economic) to implement them. To date, the rate of growth of the support for climate change resilience lags the rapid growth of cascading and converging risks. Greater emphasis on adaptation is now needed.

We urge that national leaders at COP27 pledge and take action to greatly raise the global investment in intellectual and financial resources required to support adaptation especially for the most vulnerable populations.

More details of the RACC and its International Advisory Committee are set out at https://www.stsforum.org/racc2022/iac/.
